# The Attenuating Effect of Low-Intensity Pulsed Ultrasound on Hypoxia-Induced Rat Chondrocyte Damage in TMJ Osteoarthritis Based on TMT Labeling Quantitative Proteomic Analysis

**DOI:** 10.3389/fphar.2021.752734

**Published:** 2021-12-14

**Authors:** Sa Du, Chao Liang, Yujie Sun, Bowen Ma, Wenmo Gao, Wei Geng

**Affiliations:** Department of Dental Implant Center, Beijing Stomatological Hospital, School of Stomatology, Capital Medical University, Beijing, China

**Keywords:** low-intensity pulsed ultrasound, hypoxia, chondrocyte, temporomandibular joint osteoarthritis, tandem mass tag-labeling proteomics, biomarker

## Abstract

Temporomandibular joint osteoarthritis (TMJOA) is a degenerative disease with a complex and multifactorial etiology. An increased intrajoint pressure or weakened penetration can exacerbate the hypoxic state of the condylar cartilage microenvironment. Our group previously simulated the hypoxic environment of TMJOA *in vitro*. Low-intensity pulsed ultrasound (LIPUS) stimulation attenuates chondrocyte matrix degradation *via* a hypoxia-inducible factor (HIF) pathway-associated mechanism, but the mode of action of LIPUS is currently poorly understood. Moreover, most recent studies investigated the pathological mechanisms of osteoarthritis, but no biomarkers have been established for assessing the therapeutic effect of LIPUS on TMJOA with high specificity, which results in a lack of guidance regarding clinical application. Here, tandem mass tag (TMT)-based quantitative proteomic technology was used to comprehensively screen the molecular targets and pathways affected by the action of LIPUS on chondrocytes under hypoxic conditions. A bioinformatic analysis identified 902 and 131 differentially expressed proteins (DEPs) in the <1% oxygen treatment group compared with the control group and in the <1% oxygen + LIPUS stimulation group compared with the <1% oxygen treatment group, respectively. The DEPs were analyzed by gene ontology (GO), KEGG pathway and protein-protein interaction (PPI) network analyses. By acting on extracellular matrix (ECM)-associated proteins, LIPUS increases energy production and activates the FAK signaling pathway to regulate cell biological behaviors. DEPs of interest were selected to verify the reliability of the proteomic results. In addition, this experiment demonstrated that LIPUS could upregulate chondrogenic factors (such as Sox9, Collagen Ⅱ and Aggrecan) and increase the mucin sulfate content. Moreover, LIPUS reduced the hydrolytic degradation of the ECM by decreasing the MMP3/TIMP1 ratio and vascularization by downregulating VEGF. Interestingly, LIPUS improved the migration ability of chondrocytes. In summary, LIPUS can regulate complex biological processes in chondrocytes under hypoxic conditions and alter the expression of many functional proteins, which results in reductions in hypoxia-induced chondrocyte damage. ECM proteins such as thrombospondin4, thrombospondin1, IL1RL1, and tissue inhibitors of metalloproteinase 1 play a central role and can be used as specific biomarkers determining the efficacy of LIPUS and viable clinical therapeutic targets of TMJOA.

## Introduction

Temporomandibular joint osteoarthritis (TMJOA), which is characterized by cartilage degradation, subchondral bone remodeling and redundant bone formation, constitutes a common group of clinical diseases and is an important subtype of temporomandibular joint disorder (TMD) (Stegenga, 2001; Yang et al., 2017). Rotation of the joint causes synovial fluid to flow over the surface of the condylar cartilage, which results in providing nutrients and oxygen to this cartilage. The oxygen concentrations at the surface and deep inside the joint are approximately 7% and less than 1%, respectively (Jahr et al., 2019). Chondrocytes are the only cells present in the condylar tissue of the joint. The hypoxic state of the survival microenvironment is further exacerbated in the setting of osteoarthritis (OA), which is characterized by almost no oxygen tension deep in the joint (James et al., 1990; Pfander and Gelse, 2007; Hsieh et al., 2010). It has been suggested that the intraarticular pressure exceeds the peripheral vascular hydrostatic pressure in arthritis, which results in the creation of a hypoxic environment in the joint cavity. Hypoxia-reperfusion injury can produce free radicals that damage joint tissue and increase inflammation within the joint (Nitzan, 1994; Yamaza et al., 2004). In addition, mandibular condylar cartilage has been suggested to be an avascular structure that is nutritionally dependent on penetration. When the joint is subjected to excessive stress loads, the penetration is diminished, which leads to intraarticular hypoxia (Huang et al., 2017). This observation suggests that hypoxia and inflammation promote each other and play an important role in the TMJOA process. However, the specific effects of hypoxia and the molecular pathways involved in this process remain relatively unclear.

Condylar articular cartilage has a limited ability for self-repair (Tanaka et al., 2015; Wang et al., 2015), and the current treatments are mainly causative agents, including occlusal factors or psychosomatic factors, which cannot directly act on the damaged part of the joint. Thus, a treatment method that can directly act locally at the injury site and effectively promote cartilage tissue damage repair is urgently needed (Wang et al., 2015). Low-intensity pulsed ultrasound (LIPUS) outputs pulsed waves with an intensity of less than 100 mW/cm^2^. This mechanical energy is transmitted to cells or tissues through the intervening medium with minimal thermal effects. LIPUS has the advantages of easy operation, low cost, and excellent targeting and has considerable clinical potential (Jiang et al., 2019; Tanaka et al., 2020).

Recently, LIPUS has been used to treat fractures because it can accelerate fracture healing (Poolman et al., 2017). In addition, LIPUS has been shown to exhibit evident efficacy in nerve injury recovery, the acceleration of soft tissue regeneration, and the promotion of tissue microcirculation (Jiang et al., 2019; Uddin and Komatsu, 2020). Our group found that LIPUS reduces the inflammation level in condylar cartilage, attenuates matrix hydrolysis, and inhibits osteoclastic function in rats with chronic sleep deprivation (Liang et al., 2019; Liang et al., 2020). With an intensity of 45 mW/cm^2^, the optimal effect is achieved after at least 2 weeks of treatment (Liang et al., 2020). Moreover, *in vitro* simulation of a hypoxic environment has revealed that LIPUS can slow cartilage damage by upregulating HIF-1α and downregulating the HIF-2α pathway (Yang et al., 2020). Current studies of cartilage, synovial fluid, serum and urine associated with joint disease utilizing proteomics aim to understand the pathogenesis and progression of arthritis (Hsueh et al., 2014). However, the exploration of biomarkers for the therapeutic effects of LIPUS against OA remains a research gap, which limits the early application of LIPUS in the clinical treatment of TMJOA. Biomarkers that can comprehensively identify the specific and sensitive effects of LIPUS interventions are still lacking and needed to make the treatment of TMJ-OA early, safe and controllable.

To satisfy these fundamental unmet needs regarding the treatment of OA with LIPUS, proteomics technologies have great potential for the discovery of novel and specific biochemical markers. Proteomics based on liquid chromatography-tandem mass spectrometry (LC-MS/MS) is a powerful tool for the identification and quantification of proteins in biological samples (Li and Zhu, 2020). Mass spectrometry exhibits good sensitivity for small molecule analysis, which facilitates the application of LC-MS/MS for better peptide quantification (Rauh, 2012). This experiment utilized tandem mass tag (TMT) labeling quantitative proteomic technology to analyze and compare the differentially expressed proteins (DEPs) in chondrocytes after treatment with 5% O_2_, 1% O_2_, and the combination of 1% O_2_ and LIPUS. This study aimed to identify the key proteins and pathways affected by LIPUS, screen for reliable molecular markers and provide more precise guidance for the application of LIPUS in the clinic. Moreover, several biological functions related to the FAK pathway, such as viability, migration, matrix production and degradation (Cheng et al., 2014; Jang et al., 2014; Zhang et al., 2016; Sang et al., 2020), were selected to validate the therapeutic effect of LIPUS on chondrocyte hypoxic injury.

## Materials and Methods

### Cell Isolation and Grouping

Our experiments were conducted in accordance with biosafety standards and institutional safety procedures and obtained ethical approval by Beijing Stomatological Hospital (review number KQYY-201610–001).

Primary temporomandibular condylar chondrocytes were isolated from 3-week-old healthy male Wistar rats (SPF Biotechnology, Beijing, China). The steps for the isolation and culture of these cells were based on a procedure previously published by our group (Yang et al., 2020). Third-generation chondrocytes were incubated in six-well plates (Corning, United States) in the following three groups: normal group (N group), cultured in an atmosphere containing 5% oxygen; low oxygen tension group (L group), cultured in an atmosphere containing less than 1% oxygen; and low oxygen tension + LIPUS treatment group (LP group), incubated in an atmosphere containing less than 1% oxygen and subjected to LIPUS stimulation. Chondrocytes were cultured for 24 h, followed by 20 min of LIPUS stimulation.

### LIPUS Treatment

**FIGURE 1:**
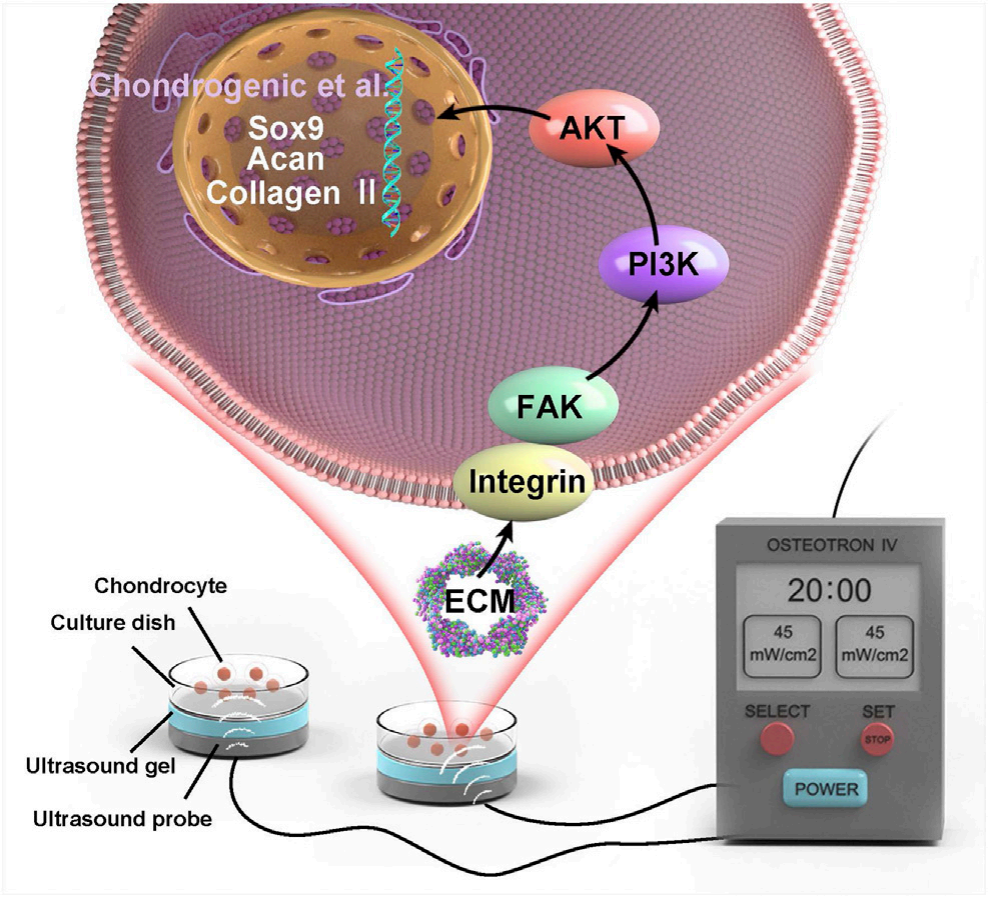
Schematic diagram of LIPUS stimulated chondrocytes.

An OSTEOTRON ultrasound device (ITO Ultrashort Wave Co. Ltd., Tokyo, Japan) was set up with the following parameters: intensity, 45 mW/cm^2^; frequency, 1.0 MHz; pulse width, 200 µs; and duty cycle, 20%. The probe of the ultrasound transmitter was placed at the bottom of the six-well plate, and the sham ultrasound transmitter was placed in the wells of the N and L groups. Ultrasound gel (Aquasonic 100, America) was applied between the probe and the bottom of the six-well plate (Figure 1). LIPUS stimulation was applied for 20 min daily for 3 consecutive days.

### TMT-Based Quantitative Proteomic Analysis

#### TMT Labeling and Fractionation

Firstly, the chondrocytes were stimulated with LIPUS for 20 min per day for three consecutive days. On the third day, cell samples from each group were added to lysis solution (8 M urea and 1% protease inhibitor cocktail) and lysed by ultrasonication. The supernatant was collected after centrifugation. Proteins were denatured with 5 mM dithiothreitol for 30 min at 56°C, alkylated with 11 mM iodoacetamide for 15 min at room temperature in the dark and then diluted to obtain a urea concentration of less than 2 M by the addition of 100 mM TEAB. Subsequently, trypsin was added at a trypsin:protein mass ratio of 1:50 for the first digestion, which was performed overnight, and at a trypsin:protein mass ratio of 1:100 for the second digestion, which was performed for 4 h. After trypsin digestion, the peptides were desalted on a Strata X C18 SPE column (Phenomenex) and dried under vacuum. The peptides were reconstituted in 0.5 M TEAB and processed with the TMT Mass Tagging Kit (Thermo Fisher, United States) according to the manufacturer’s protocol. In this study, labeling reagents 126, 127N, 127C, 128N, 128C 129N, 129C, 130N and 131 were used to label three biological replicates of the N, L and LP groups. The samples were then fractionated by high-pH reversed-phase HPLC using a Thermo Betasil C18 column (Thermo Fisher, United States).

#### Liquid Chromatography–Tandem Mass Spectrometry Analysis

The peptides were dissolved in LC mobile phase A and then separated using an EASY-nLC 1,000 ultrahigh-performance liquid chromatograph (Thermo Fisher, United States). Mobile phase A was an aqueous solution containing 0.1% formic acid and 2% acetonitrile, and mobile phase B was an aqueous solution containing 0.1% formic acid and 90% acetonitrile. The gradient settings for the liquid phase were as follows: 0–40 min, 7 ∼ 25% B; 40–52 min, 25 ∼ 35% B; 52–56 min, 35 ∼ 80% B; and 56–60 min, 80% B. The flow rate was maintained at 500.00 nL/min. The peptides were exposed to a nanospray ionization (NSI) source and analyzed by tandem mass spectrometry (MS/MS) with a Q Exactive™ Plus mass spectrometer (Thermo Fisher, United States) coupled online to the UPLC system. The applied electrospray voltage was 2.1 kV. For full scans, the primary MS scan range was set to 400–1,500 m/z, and intact peptides were detected using an Orbitrap with a resolution of 70,000. Peptides were then selected for MS/MS with a normalized collision energy (NCE) setting of 28, and the secondary scan resolution was set to 35,000.00. The MS/MS scan range was fixed to 100 m/z. The data acquisition mode used a data-dependent acquisition (DDA) program. The top 20 peptide parent ions with the highest signal intensity were selected to enter the higher-energy collision dissociation (HCD) collision cell sequentially after the primary scan and were fragmented using a fragmentation energy of 28, 32%. The automatic gain control (AGC) was set to 5E4. The signal threshold was set to 3.8E4 ions/s. The dynamic exclusion duration of the MS/MS scan was set to 30 s. The protein mass spectrometry analysis was performed by PTM Biolabs, Inc. (Hangzhou, China).

#### TMT-Based Protein Identification and Quantification

MS/MS spectra were retrieved using MaxQuant (v1.6.15.0). The search database was *Rattus norvegicus* 10,116, and the reverse decoy database was added to calculate the false discovery rate (FDR) due to random matching. A common contamination library was added to the database to eliminate the effects of contaminating proteins on the identification results. Trypsin/P was specified as the cleavage enzyme, and up to 2 missing cleavages were allowed. The minimum peptide length was set to 7 amino acid residues. The maximum number of peptide modifications was set to 5. The mass tolerance of the precursor ions was set to 20 ppm for the first search and 5 ppm for the main search. The mass tolerance of secondary fragment ions was set to 20.0 ppm. Carbamidomethyl modification on Cys was set as the fixed modification, and acetylation modification and oxidation on Met were set as variable modifications. The quantification method was set to TMT-10plex. The FDR for protein and PSM identification was set to 1%. If the *p*-value was <0.05, an expression fold change greater than 1.3 was considered to indicate significant upregulation, whereas a fold change less than 1/1.3 was considered to indicate significant downregulation.

#### Bioinformatic Analyses

Gene ontology (GO) functional annotation based on the DAVID bioinformatics database (https://david.ncifcrf.gov/gene2gene.jsp) was first performed. The identified proteins were divided into the following three categories: biological process (BP), cellular component (CC), and molecular function (MF). The DEPs were further divided into up- and downregulated groups and analyzed for significant functional enrichment.

Kyoto Encyclopedia of Genes and Genomes (KEGG) pathway enrichment was performed using the KEGG database (https://www.genome.jp/kegg/pathway.html) for preformed network queries. The proteins were classified according to the online KEGG pathway hierarchical classification method and were then subjected to enrichment analysis.

Enrichment analyses of GO and KEGG annotations were performed using Fisher’s exact test for significance analysis.

Protein-protein interaction (PPI) information for the DEPs was obtained using STRING online software (https://string-db.org/). Cytoscape software (National Resource for Network Biology, United States) was used to generate network diagrams. The MCODE plugin was then used to mine the network for modules with high connectivity.

### CCK8 Assay

CCK8 reagent (Dojindo, Japan) was diluted 10-fold with serum-free high-glucose DMEM in 96-well plates (Corning, United States) seeded at 3,000 cells/well. After 24 h of daily LIPUS stimulation, 100 μL of the diluted CCK8 reagent was added to each well. The OD values were measured at 450 nm using an enzyme marker (Thermo Fisher, United States) after 2 h of incubation.

### Wound Healing Assay

Condylar chondrocytes were inoculated in a six-well plate at 5 × 10^5^ cells/well. Once the cells reached 90% confluence on the bottom of the plate, the cell layer was scratched from one side of the well to the other with a 200-μL pipette tip and observed continuously for 72 h. Specifically, the cells were observed under a microscope with a 4× objective (OLYMPUS, Japan) at 0, 24, 48, and 72 h. The migration distance was calculated using ImageJ by dividing the unfilled scratch area in a single field of view by the height of the field of view. The migration rate was calculated as follows: migration rate = scratch width reduced at the time point of interest/scratch width at 0 h.

### Alcian Blue Staining

Condylar chondrocytes were inoculated in six-well plates at a density of 5 × 10^5^ cells/well. LIPUS stimulation was performed for three consecutive days. After 3 h of LIPUS stimulation on the third day, the cells were fixed with methanol, rinsed 3 times with distilled water and stained with Alcian blue staining solution (Solarbio, China) for 30 min. The staining solution was discarded, and the cells were rinsed, observed and scanned.

### Real-Time Quantitative Polymerase Chain Reaction

Cells were seeded at an intensity of 5 × 10^5^ cells/well in 6-well plates. LIPUS treatment was carried out for three continuous days. After 3 h of LIPUS stimulation on the third day, total RNA was obtained using TRIzol reagent and an Ultrapure RNA Kit (CWbiotech, China). cDNA was obtained by reverse transcription using a Super RT cDNA Synthesis Kit (CWbiotech, China) and then was amplified by RT-qPCR using UltraSYBR mix. In this study, *β-actin* was used as the housekeeping gene and 2^−ΔΔCT^ was used to calculate the relative change in gene expression. The primer sequences used to amplify *Sox9*, *Collagen Ⅱ*, *MMP3*, *TIMP1*, *Aggrecan* (*Acan*), *Vegfa*, *thrombospondin4* (*Thbs4*), *thrombospondin1* (*Thbs1*), *Il1rl1*, *Glul* and *β-actin* are listed in Table 1.

**TABLE 1:** Primer sequences used for RT-qPCR.

Gene	Primer sequence (5′ to 3′)
*Sox9*	Forward: GAC​GTG​CAA​GCT​GGG​AAA​GT
	Reverse: CGG​CAG​GTA​TTG​GTC​AAA​CTC
Collagen Ⅱ	Forward: AAGAGCAAGGAGAAGAAG
	Reverse: TTACAGTGGTAGGTGATG
*MMP3*	Forward: GCT​CAC​TTT​GAT​GAT​GAT​GAA​CGA​TGG
	Reverse: TGT​GGA​GGA​CTT​GTA​GAC​TGG​GTA​C
*TIMP1*	Forward: CGA​GAC​CAC​CTT​ATA​CCA​GCG​TTA​TG
	Reverse: CGG​TTC​TGG​GAC​TTG​TGG​ACA​TAT​C
*Aggrecan*	Forward: AGA​AGA​GAC​CCA​AAC​AGC​AGA​AAC​AG
	Reverse: GCA​GGT​GGC​TCC​ATT​CAG​ACA​AG
*Vegfa*	Forward: AAC​CTC​ACC​AAA​GCC​AGC​ACA​TAG
	Reverse: GAC​CCT​TTC​CCT​TTC​CTC​GAA​CTG
*Thbs4*	Forward: GAC​TCC​TGT​GAC​ACC​AAC​CAA​GAC
	Reverse: TCA​TCG​CAT​TCA​TCG​CCA​ATC​CC
*Thbs1*	Forward: ACC​GAC​GGG​ACA​CAC​GAC​TG
	Reverse: TGA​TGC​CAT​TGC​CTG​CGT​AGC
*Il1rl1*	Forward: GCT​TTG​GGC​TTT​GGC​AAT​TCT​GAC
	Reverse: GGG​TTA​ATC​GCA​CCT​CCT​CTT​TGG
*Glul*	Forward: TCC​ACG​AAA​CCT​CCA​ACA​TCA​ACG
	Reverse: CGG​TCT​TCA​AAG​TAA​CCC​TTC​TTC​TCC
*β-actin*	Forward: ATG​TGG​ATC​AGC​AAG​CAG​GA
	Reverse: GGT​GTA​AAA​CGC​AGC​TCA​GTA​A

### Western Blot (WB) Analysis

The cell seeding density and the timing of sample extraction were consistent with the PCR steps. Cells were lysed with RIPA buffer containing PMSF and a protease inhibitor cocktail (PIC) (Sigma, United States) (buffer:PMSF:PIC ratio of 100:1:1) on ice, and the protein concentration was determined using the Bradford method. Proteins were separated by 10% SDS polyacrylamide gel electrophoresis (Bio-Rad, United States) and transferred to PVDF membranes (Bio-Rad, United States) using a semidry transfer apparatus (Bio-Rad, United States). The membranes were incubated with the primary antibody overnight at 4°C and with a horseradish peroxidase-conjugated anti-rabbit secondary antibody (ABclonal, China) for 1 h. The immunoreactions were visualized using ECL reagents (Bio-Rad, United States) and imaged with a ChemiDoc MP Imaging System (Bio-Rad, United States). The primary antibodies included rabbit polyclonal anti-collagen Ⅱ (1:3,000, ab34712, Abcam), rabbit monoclonal anti-aggrecan (1:1,000, A11691, ABclonal), rabbit polyclonal anti-MMP3 (1:1,000, ab52915, Abcam), rabbit polyclonal anti-TIMP1 (1:1,000, ab61224, Abcam), rabbit polyclonal anti-VEGFA (1:1,000, A12303, ABclonal), rabbit polyclonal anti-THBS4 (1:1,000, ab263898, Abcam), rabbit monoclonal anti-THBS1 (1:1,000, ab22928-10, Abcam), rabbit polyclonal anti-IL1RL1 (1:1,000, ab228543, Abcam), rabbit polyclonal anti-GLUL (1:1,000, ab176562, Abcam), and anti-β-actin (1:100,000, AC026, ABclonal) antibodies.

### Statistical Analysis

All data were statistically analyzed with SPSS 26.0 software, and all charts were generated using GraphPad Prism 6.0. Each experiment was repeated more than three times. One-way analysis of variance (ANOVA) or Student’s *t-test* was used for the assessment of statistical significance. Significance was defined at three levels (*p < 0.05, **p < 0*.*01, and ***p < 0.001).

## Results

### Quantitative Identification of DEPs

**FIGURE 2:**
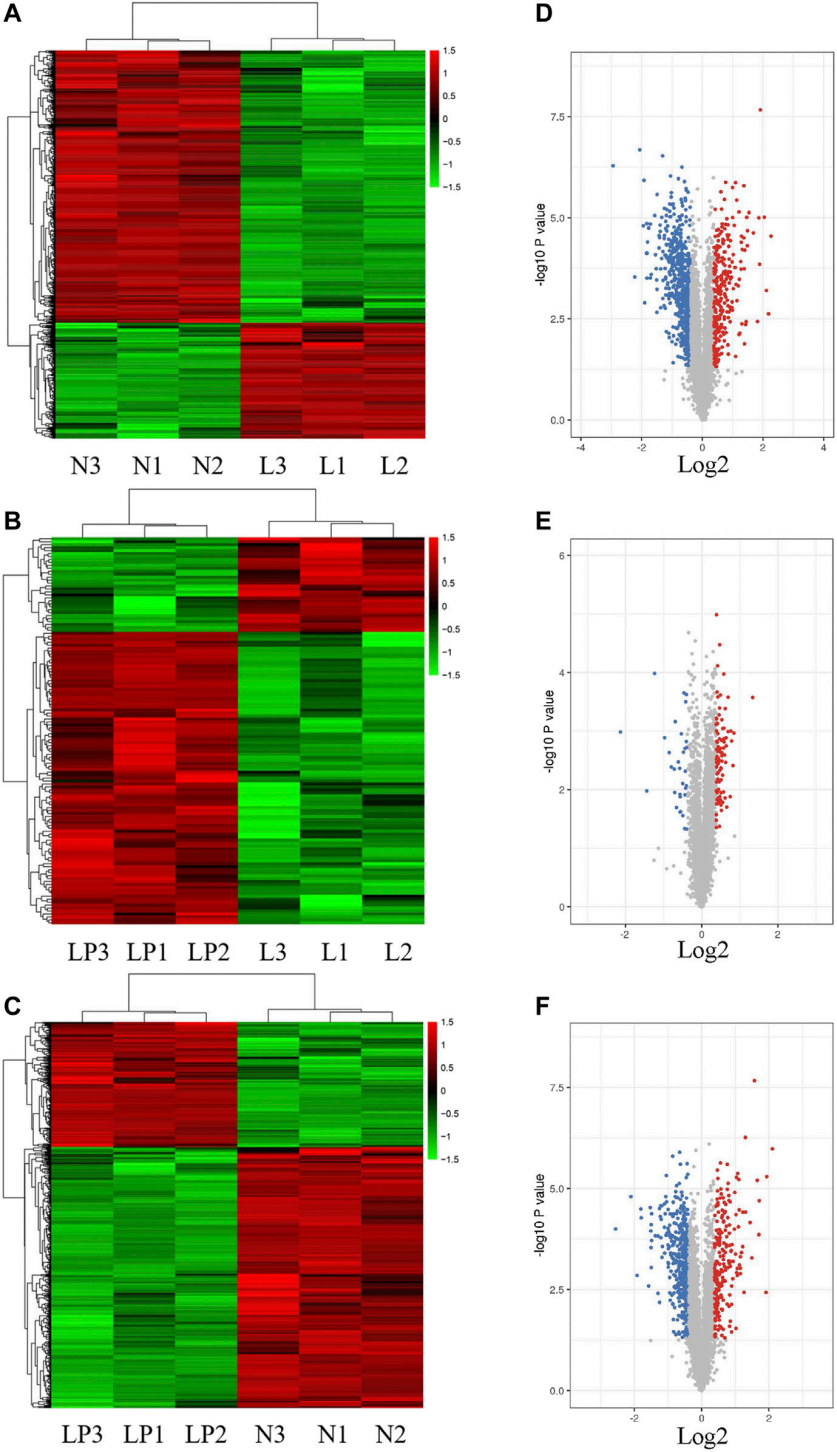
Heat maps and volcano plots showing the significantly differentially expressed proteins. Heat maps of DEPs identified from the L/N **(A)**, LP/L **(B)** and LP/N **(C)** comparisons. Volcano plots of DEPs identified from the L/N **(D)**, LP/L **(E)** and LP/N **(F)** comparisons. The red dots represent upregulated proteins, and the blue dots represent downregulated proteins.

Supplementary Figure S1A shows the cultured primary chondrocytes and third-generation cells identified by Alcian blue staining (Supplementary Figure S1B). The numbers of total spectra, matched spectra, peptides, unique peptides, identified proteins and quantifiable proteins in this experiment are shown in Supplementary Figure S2A. A total of 5,587 proteins were identified, and 4,480 of these proteins were quantified. All DEPs in the three groups are summarized in Table 2. As illustrated in the heat maps (Figures 2A–C) and volcano plots (Figures 2D–F), 902 DEPs (269 upregulated and 633 downregulated DEPs) were identified between the L and N groups, 131 DEPs (99 upregulated and 32 downregulated DEPs) were identified between the LP and L groups, and 657 DEPs (213 upregulated and 444 downregulated DEPs) were identified between the LP and N groups. The relative standard deviation (RSD) assay showed good reproducibility of the quantification for all three samples in each group (Supplementary Figure S2B).

**TABLE 2:** Numbers of up/downregulated DEPs between the three groups.

Compared groups	Upregulated	Downregulated	Total difference
L/N	269	633	902
LP/L	99	32	131
LP/N	213	444	657

### GO Enrichment Analysis

**FIGURE 3:**
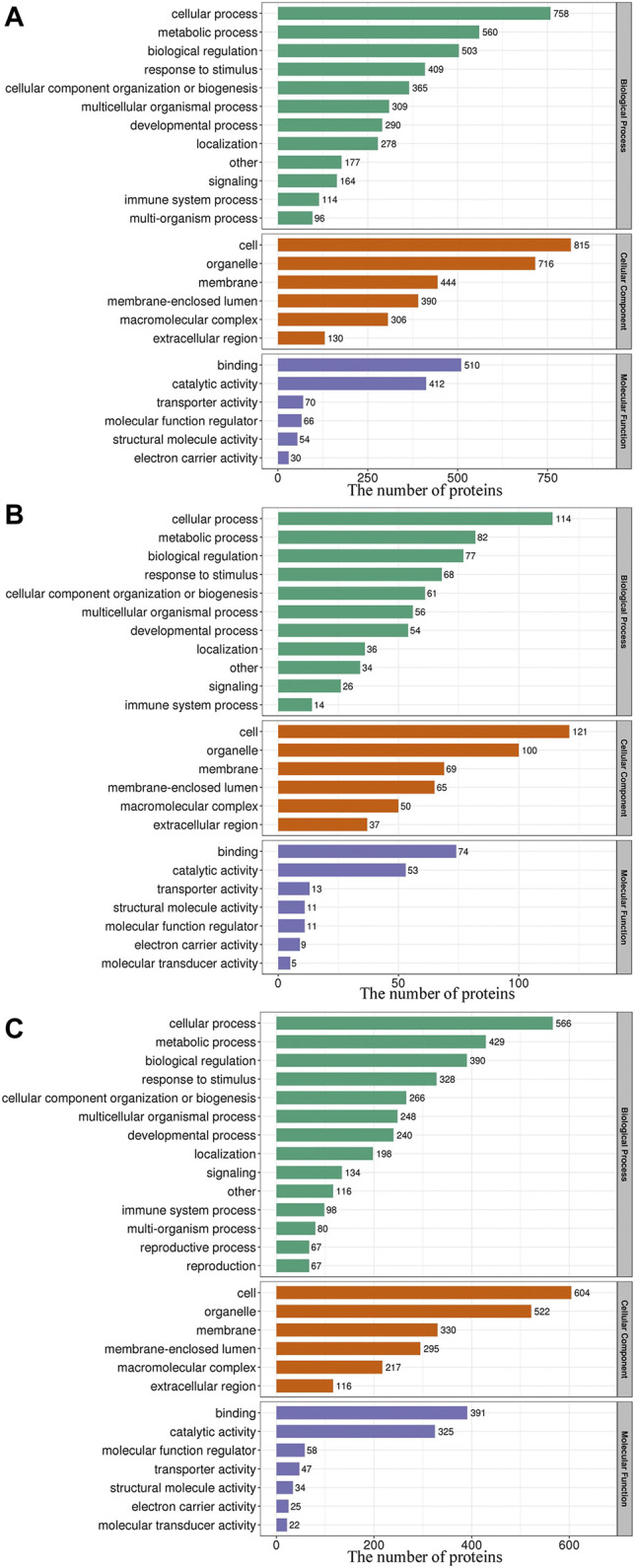
GO functional classification of the DEPs. The 3 bar charts show the GO functional classification of the DEPs identified from the L/N **(A)**, LP/L **(B)** and LP/N **(C)** comparisons.

As shown in Figure 3, within the GO BP category, the DEPs were enriched mainly in the terms cellular process, metabolic process and biological regulation, and 45.26, 43.89, and 42.92% of the DEPs identified from the L/N, LP/L and LP/N comparisons, respectively, were enriched in these terms. The analysis of the CC category revealed that the DEPs were distributed mainly in cells, organelles and cell membranes, and 70.51, 65.61 and 69.87% of the DEPs identified from the L/N, LP/L and LP/N comparisons, respectively, were enriched in these terms. In the MF category, the DEPs were related mainly to binding and catalytic activity, and these terms were found for 80.74, 72.16 and 79.38% of the DEPs identified from the L/N, LP/L and LP/N comparisons, respectively.

**FIGURE 4:**
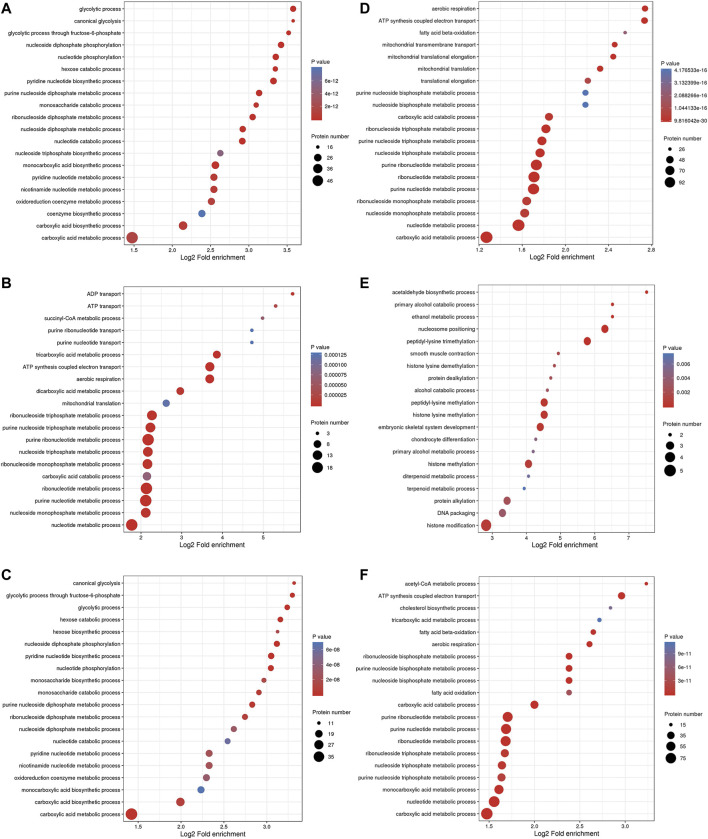
GO enrichment analysis of DEPs. Enrichment of the BP category with upregulated DEPs identified from the L/N **(A)**, LP/L **(B)** and LP/N **(C)** comparisons and downregulated DEPs identified from the L/N **(D)**, LP/L **(E)** and LP/N **(F)** comparisons.

The BP enrichment analysis showed that the upregulated DEPs identified from the L/N comparison were mainly enriched in the terms glycolysis process, canonical glycolysis and glycolysis process through fructose-6-phosphate (Figure 4A), and the downregulated DEPs were mainly enriched in the terms aerobic respiration, ATP synthesis coupled electron transport, and fatty acid β-oxidation (Figure 4D). The upregulated DEPs identified from the LP/L comparison were mainly enriched in the terms ADP transport, ATP transport, and succinyl-CoA metabolism (Figure 4B), and the downregulated DEPs were mainly enriched in the terms acetaldehyde biosynthesis process, primary alcohol catabolic process, and ethanol metabolism process (Figure 4E). The upregulated DEPs obtained from the LP/N comparison were mainly enriched in the terms canonical glycolysis, glycolysis process through fructose-6-phosphate and glycolytic process (Figure 4C), and the downregulated DEPs were mainly enriched in the terms acetyl-CoA metabolic process, ATP synthesis coupled electron transport, and cholesterol biosynthesis process (Figure 4F). Supplementary Figure S3A–C shows the enrichment of the CC category with the upregulated DEPs in the three groups, and Supplementary Figure S3D–F shows the enrichment of this category with the downregulated DEPs. Supplementary Figure S4A–C shows the enrichment of the MF category with the upregulated DEPs in the three groups, and Supplementary Figure S4D–F shows that with the downregulated DEPs. The subcellular localization of the upregulated DEPs in the three groups is shown in Supplementary Figure S5A–C, and that of the downregulated DEPs is shown in Supplementary Figure S5D–F.

### Kyoto Encyclopedia of Genes and Genomes Analysis

**FIGURE 5:**
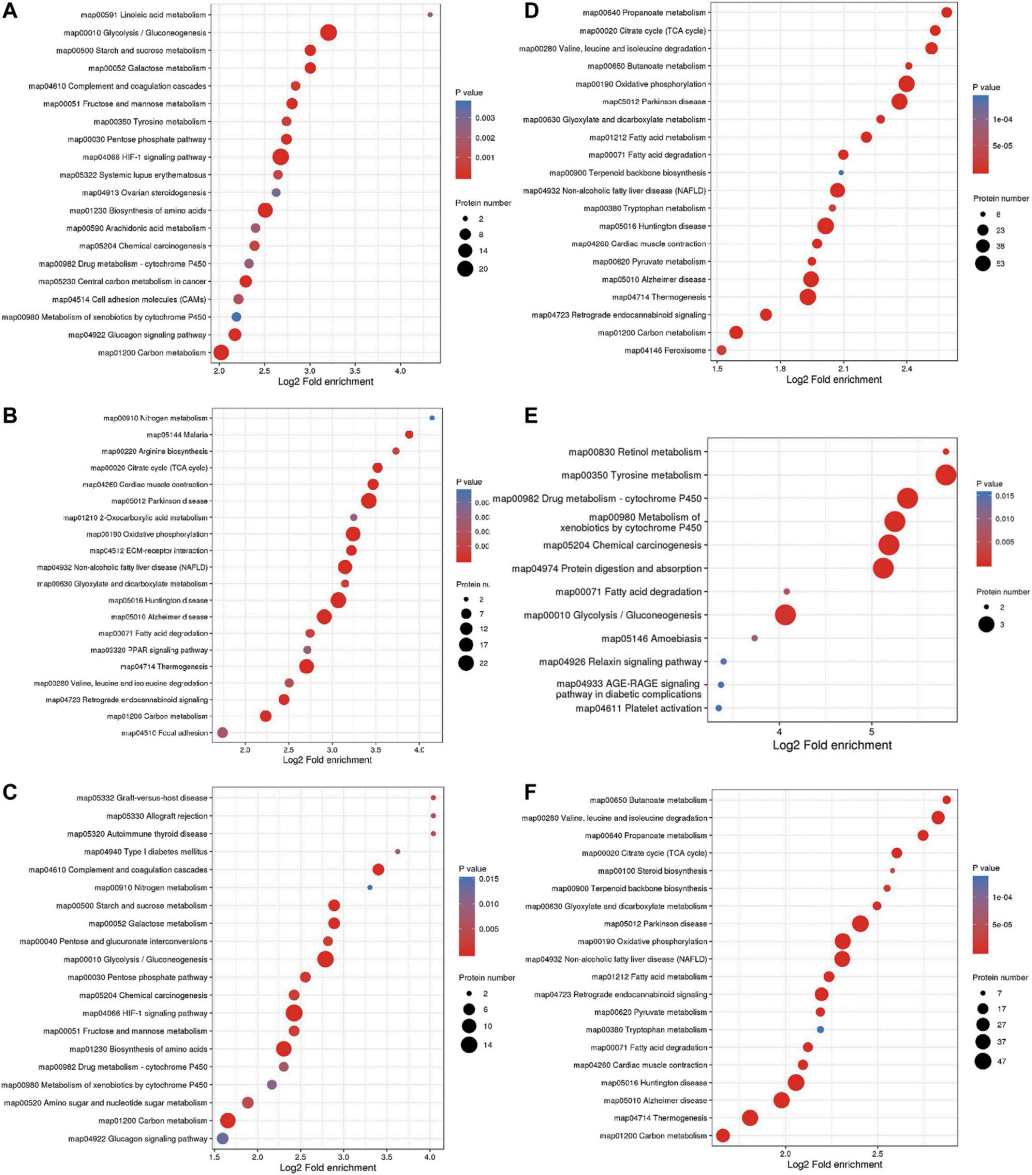
KEGG pathway enrichment analysis of DEPs. Enrichment of KEGG pathways with upregulated DEPs identified from the L/N **(A)**, LP/L **(B)** and LP/N **(C)** comparisons and downregulated DEPs identified from the L/N **(D)**, LP/L **(E)** and LP/N **(F)** comparisons.

**FIGURE 6:**
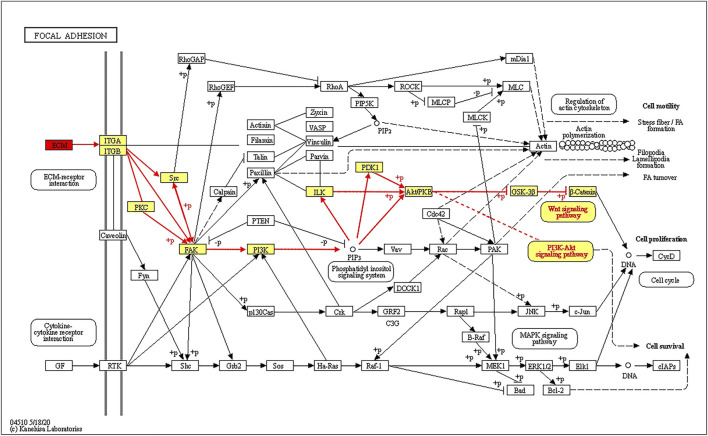
Schematic diagram of the FAK signaling pathway showing that ECM proteins can gradually regulate the ITGA/B-FAK-PI3K-AKT signaling pathway.

The top three pathways enriched with upregulated DEPs were linoleic acid metabolism (20.07-fold, *p <* 0.01), glycolysis/gluconeogenesis (9.23-fold, *p* < 0.001), and starch and sucrose metabolism (8.03-fold, *p* < 0.001) in addition to the HIF-1 signaling pathway (6.4-fold, *p* < 0.001) (Figure 5A). The top three pathways enriched with downregulated DEPs were propanoate metabolism (6.01-fold, *p* < 0.001), citrate cycle (TCA cycle) (5.79-fold, *p* < 0.001), and valine, leucine and isoleucine degradation (5.72-fold, *p* < 0.001) (Figure 5D). The pathways enriched with the upregulated DEPs identified from the LP/L comparison were nitrogen metabolism (17.71-fold, *p* < 0.05), malaria (14.76-fold, *p* < 0.001), and arginine biosynthesis (13.29-fold, *p* < 0.01) as well as the focal adhesion pathway (3.34-fold, *p* < 0.01) and PI3K-Akt pathway (2.83-fold, *p* < 0.01) (Figure 5B). The pathways enriched with the downregulated DEPs identified from this comparison were retinol metabolism (55.8-fold, *p* < 0.001), tyrosine metabolism (55.8-fold, *p* < 0.001), and drug metabolism - cytochrome P450 (41.85-fold, *p* < 0.001) (Figure 5E). The pathways enriched with the upregulated DEPs identified from the LP/N comparison were graft-versus-host disease (16.46-fold, *p* < 0.01), allograft rejection (16.46-fold, *p <* 0.01) and autoimmune thyroid disease (16.46-fold, *p* < 0.01) (Figure 5C), and those enriched with the downregulated DEPs obtained from this comparison were butanoate metabolism (7.33-fold, *p* < 0.001), valine, leucine, and isoleucine degradation (7.1-fold, *p* < 0.001), and propanoate metabolism (6.71-fold, *p* < 0.001) (Figure 5F). Schematic diagram of the focal adhesion pathway is downloaded from KEGG database at https://www.genome.jp/kegg/pathway.html (Figure 6). It shows several pathways connected to FAK, including PI3K-AKT pathway, Regulation of actin cytoskeleton, Wnt signaling pathway and MAPK signaling pathway.

### PPI Network Construction

**FIGURE 7:**
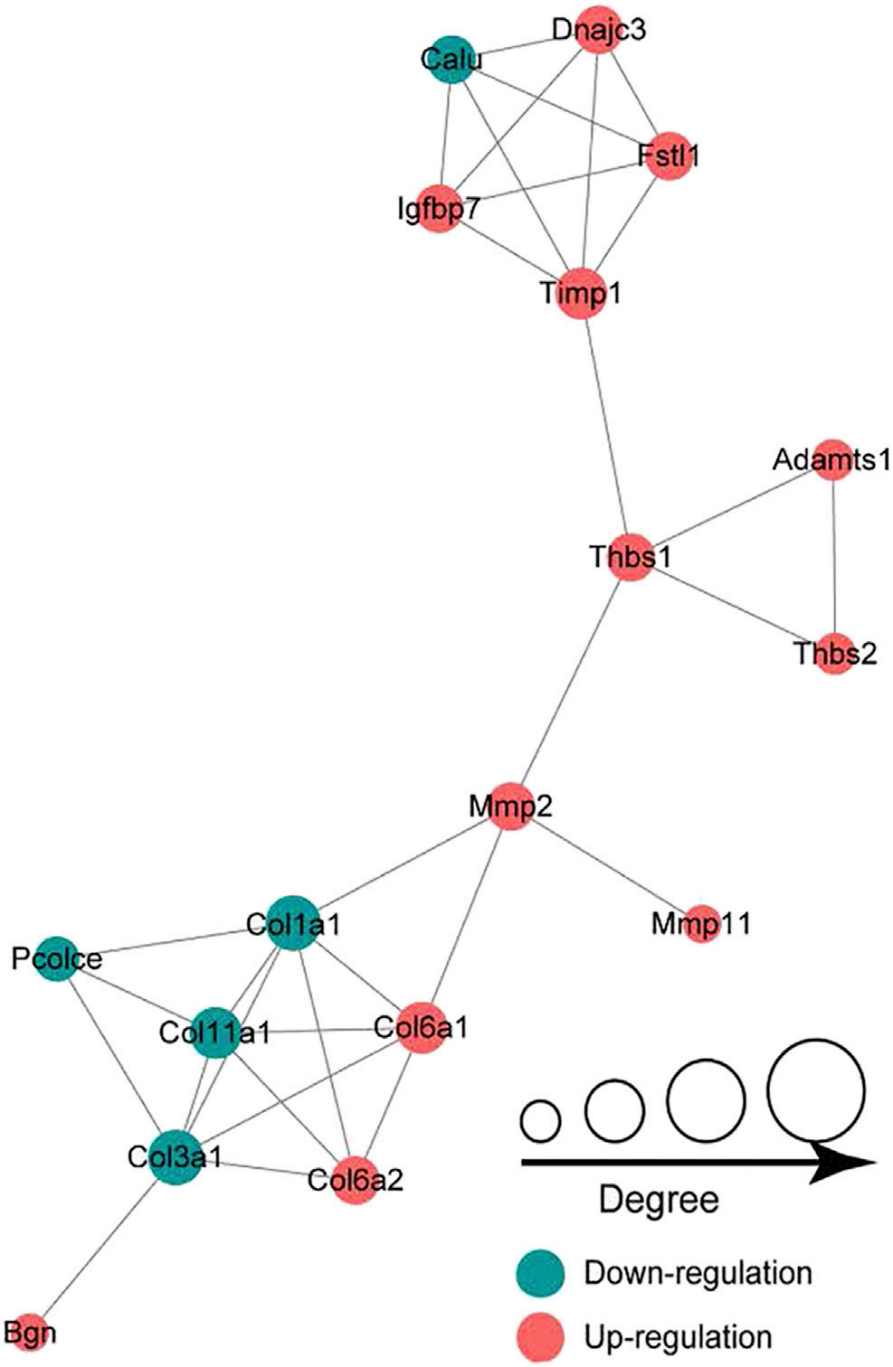
Protein-protein interaction network analysis showing that Mmp2 and Thbs1 are the key hub proteins.

In the LP/L comparison, Mmp2 (Protein accession code: P33436; connectivity degree: 4) and Thbs1 (Protein accession code: M0R979; connectivity degree: 4) were the key hub proteins (Figure 7). In addition to Thbs1, Mmp2 also interacts with Mmp11 (Protein accession code: Q499S5; connectivity degree: 1), Col1a1 (Protein accession code: P02454; connectivity degree: 6), and Col6a1 (Protein accession code: D3ZUL3; connectivity degree: 5). In addition to Mmp2, Thbs1 also interacts with Timp1 (protein accession code: P30120; connectivity degree: 5), Adamts1 (protein accession code: Q68EJ2; connectivity degree: 2) and Thbs2 (protein accession code: D4A2G6; connectivity degree: 2). Thbs1, Thbs2, Col6a1, and Col6a2 are also included in the enriched KEGG pathway of focal adhesion.

### Screening and Validation of DEPs

**FIGURE 8:**
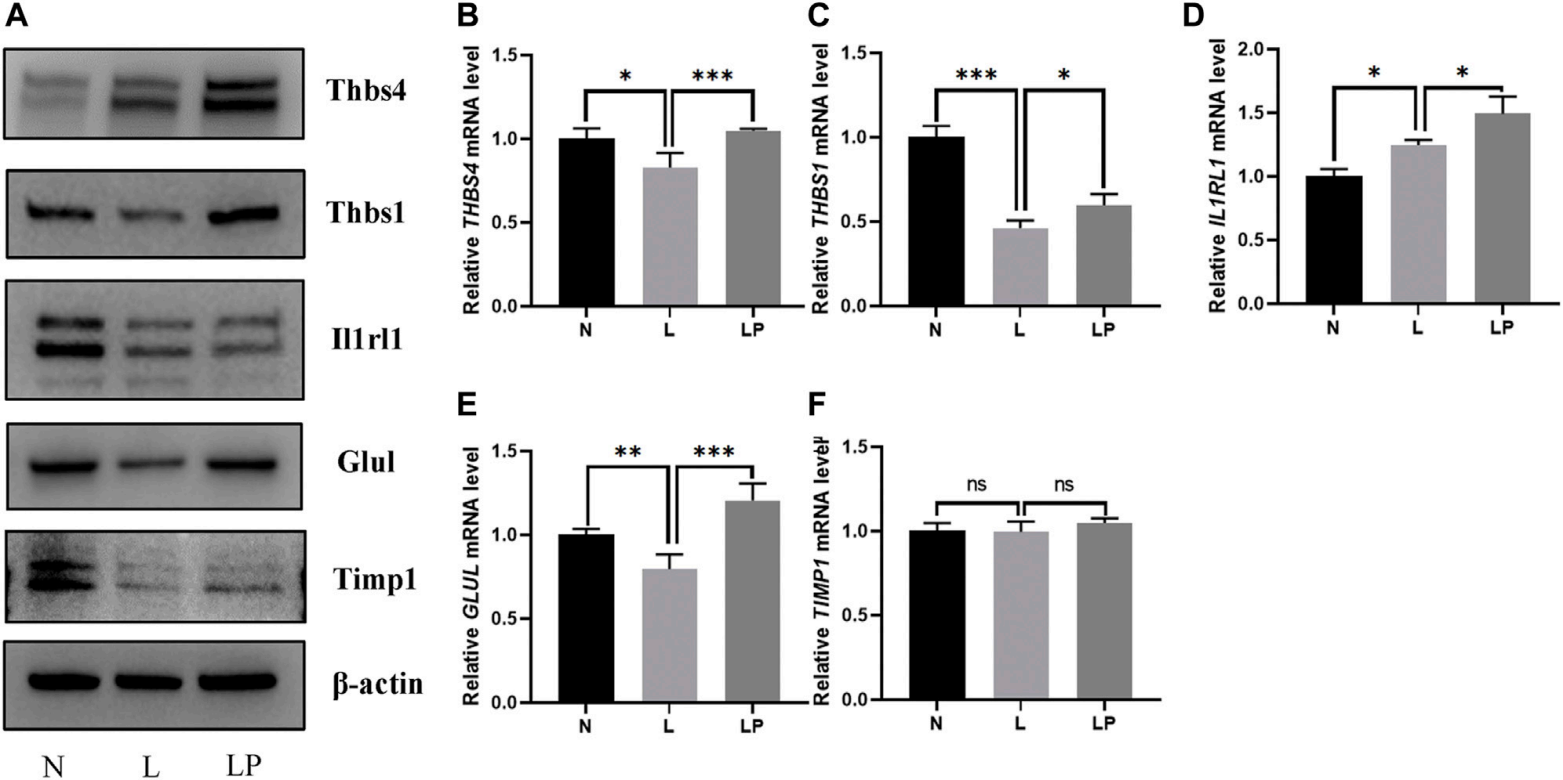
Validation of DEPs identified from the comparisons of the N, L and LP groups. **(A)** Western blot analysis of proteins including THBS4, THBS1, IL1RL1, GLUL and TIMP1. With the exception of the IL1RL1 band, all bands on the Western blot were consistent with the bioinformatic analysis results. RT-qPCR analysis of the following genes: *THBS4* **(B)**, *THBS1* **(C)**, *IL1RL1* **(D)**, *GLUL* **(E)** and *TIMP1* **(F)**. The mRNA levels of *Thbs4*, *Thbs1*, *IL1RL1*, and *Glul* were consistent with the trends observed in the bioinformatic analyses. The data are shown as the mean ± SD values; *p < 0.05, **p < 0.01, and ***p < 0.001.

The DEPs screened from each group and the DEPs with the five highest expression fold changes are shown in Table 3. In this study, we selected the top five upregulated DEPs obtained from the LP/L comparison for Western blot and RT-qPCR validation. As shown in Figure 8A, the protein levels of Thbs4, Thbs1, Glul, and TIMPl but not that of IL1RL1 were consistent with the bioinformatic analysis results. In addition, the mRNA levels of *Thbs4*, *Thbs1*, *IL1RL1*, and *Glul* were consistent with the trends observed in the bioinformatic analyses (Figures 8B–F), which indicated that the TMT-based proteomics results were reliable.

**TABLE 3:** Top 5 DEPs identified from the L/N, LP/L and LP/N comparisons.

	L/N		LP/L		LP/N	
	**Name**	**Ratio**	**Name**	**Ratio**	**Name**	**Ratio**
**Up**	Mt1	4.812	Thbs4	2.524	Ptgs2	4.306
	Vegfa	4.546	Thbs1	1.798	Thbs4	3.826
	Mt1m	4.326	Glul	1.766	Vegfa	3.774
	Ptgs2	4.12	Il1rl1	1.723	Slc2a3	3.278
	Ero1a	3.775	Timp1	1.698	Mt1	3.252
**Down**	Ptn	0.13	Poll	0.368	Ptn	0.17
	Nfil3	0.213	Mgp	0.424	Aldh1a2	0.264
	Csad	0.239	Akr1c15	0.509	Acsf2	0.286
	Ndufa9	0.26	Mt1m	0.554	Marcks	0.288
	Ndufb7	0.264	Hist1h1c	0.575	Fbln5	0.336

### LIPUS Does Not Significantly Affect Cell Viability

**FIGURE 9:**
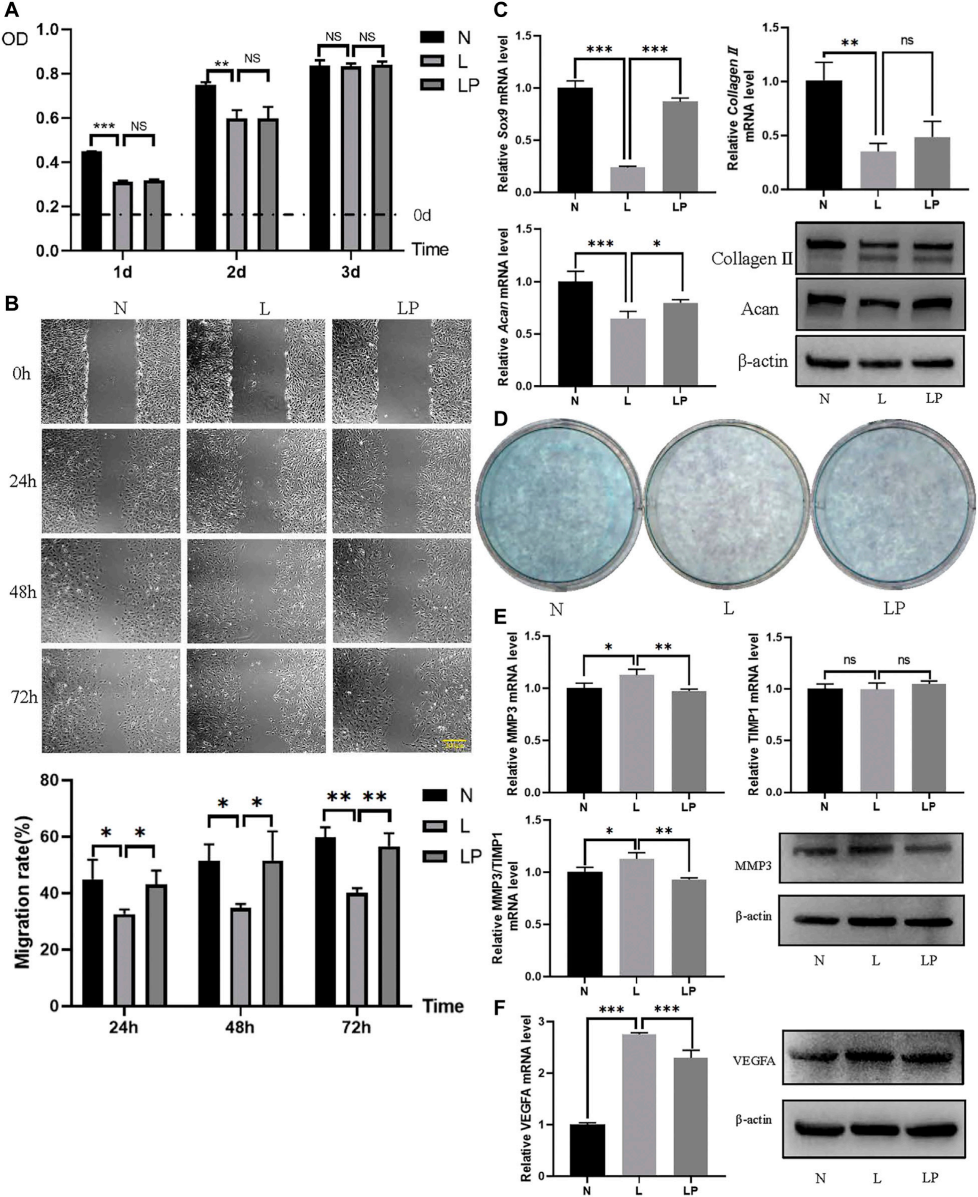
LIPUS alters the biological function of chondrocytes. **(A)** Viability of chondrocytes in the N, L and LP groups. On days 1 and 2, the number of cells in the L group was significantly lower than that in the N group. On days 1, 2 and 3, no significant difference was found between the L and LP groups. **(B)** Migration rates obtained for the N, L and LP groups. The migration rate was significantly lower in the L group than in the N group and significantly higher in the LP group than in the L group at 24, 48 and 72 h. **(C)** Comparison of the chondrogenic capacity among the N, L and LP groups. The mRNA levels of *Sox9*, *collagen II*, and *Acan* were lower in the L group than in the N group and higher in the LP group than in the L group. The protein contents of collagen II and Acan were consistent with the mRNA levels. **(D)** Alcian blue staining of mucin sulfate in chondrocytes belonging to the three groups. The lightest staining was observed in the L group. **(E)** mRNA expression levels and protein contents of MMP3 and TIMP1 in the three groups. Both the mRNA level and protein content of MMP3 appeared to be higher in the L group than in the LP and N groups. The ratio of the MMP3 to TIMP1 mRNA level appeared to be higher in the L group than in the LP group. **(F)** RT-qPCR and Western blot analyses of VEGFA. Both the mRNA level and protein content of VEGFA were higher in the L group than in the N group and lower in the LP group than in the L group. The data are shown as the mean ± SD values; *p < 0.05, **p < 0.01, and ***p < 0.001.

The number of condylar chondrocytes in the L group was significantly lower than that in the N group on days 1 and 2 and was not significantly different from that in the N group on day 3. The number of condylar chondrocytes in the LP group was not significantly different from that in the L group, which further indicated that LIPUS was not toxic to chondrocytes (Figure 9A).

### LIPUS Improves the Migration of Chondrocytes

As shown in Figure 9B, the respective migration rates obtained for the N, L and LP groups were 49.33, 30.69 and 37.40% at 24 h; 55.23, 33.26 and 39.80% at 48 h; and 59.73, 38.34 and 51.29% at 72 h. The migration rate was significantly decreased in the L group but significantly increased in the LP group.

### LIPUS Enhances the Chondrogenic Ability of Chondrocytes and Increases their Mucin Sulfate Content

The mRNA levels of Sox9 and Acan were decreased in the L group and increased in the LP group (Figure 9C). The Alcian blue staining results showed darker staining in the LP group than in the L group (Figure 9D).

### LIPUS Attenuates Matrix Hydrolysis Dysregulation and Reduces Vascularization

The mRNA level and protein content of MMP3 were significantly higher in the L group than in the LP group, but the mRNA level of *TIMP1* did not significantly differ between the L and LP groups. The Western blot analysis results showed that the TIMP1 protein level was lower in the L group than in the LP group. Moreover, the MMP3/TIMP1 ratio was significantly lower in the LP group than in the L group (Figure 9E). In addition, both the mRNA level and protein content of *VEGFA* were significantly higher in the L group than in the LP group, consistent with the bioinformatic analysis results (Figure 9F).

## Discussion

To date, studies on arthritis have mainly aimed to understand cartilage degeneration and inflammation, which also includes the search for accurate molecular markers of OA. The limited knowledge of the mechanism of action of LIPUS has hindered its clinical application. In recent decades, TMT-based proteomic analysis techniques have been used in chondrocyte studies due to their advantages of high throughput, high coverage, and ability to achieve peptide identification and quantification (Hsueh et al., 2014; Toyoda et al., 2019; Yao et al., 2019). In this study, we comprehensively screened the possible molecular targets and related pathways through which LIPUS affects chondrocytes in the presence of a 1% oxygen concentration using TMT-based quantitative proteomic technology. This finding is beneficial for the establishment of individualized LIPUS treatment plans for patients in the clinic. The reliability of the protein profile data was also verified. The results of the study revealed the identification of many DEPs and associated pathways after LIPUS treatment. Furthermore, the stress-mediated regulation of the chondrocyte survival microenvironment was observed during this process.

When the chondrocyte survival microenvironment changes, chondrocytes reprogram their metabolism to adapt to environmental changes and thereby ensure a sufficient energy supply (Zheng et al., 2021). In chondrocytes in an inflammatory microenvironment, the energy supply shifts from reliance on oxidative phosphorylation and the TCA cycle to reliance on glycolytic processes (Liu-Bryan and Terkeltaub, 2015). In this study, we performed a bioinformatic analysis and found that DEPs between the L/N groups were significantly enriched in the glycolytic process. After LIPUS stimulation, the BP category was enriched with numerous TCA-related enzymes, indicating that LIPUS can partially ameliorate the hypoxic state in chondrocytes and accelerate intracellular energy circulation and metabolism.

With the aim of screening molecular targets that are efficiency affected by LIPUS, the identification of molecular markers with high specificity and sensitivity is one of the contributions of this study. Interestingly, the DEPs identified from the LP/L comparison contained ECM proteins with high fold changes, such as thrombospondin 4 (THBS4), thrombospondin 1 (THBS1), IL1RL1, and tissue inhibitor of metalloproteinase 1 (TIMP1). Thbs4 has been shown to be sensitive to mechanical stimuli (Cingolani et al., 2011). *In vivo*, some scholars have revealed that Thbs4 is present in the articular cartilage but not in the growth plates of mice, which suggests low levels or even an absence of Thbs4 during the peak of articular cartilage development (Jeschke et al., 2015). *In vitro*, Maly concluded that Thbs4 maintains porcine chondrocyte phenotype stability by promoting collagen and proteoglycan production (Maly et al., 2021). Collagen IX has been shown to stabilize fibrous and proteoglycan networks, and Brachvogel’s study identified Thbs4 as a possible binding partner for collagen IX (Brachvogel et al., 2013). TGF-β, an important regulator of ECM remodeling, has been shown to interact with Thbs4 *in vivo* and *vitro* (Stenina-Adognravi and Plow, 2019). High levels of Thbs1 can be detected in articular cartilage and can resist vascular invasion and prevent cartilage ossification. The gene delivery of Thbs1 to rat joints inhibited the progression of OA (Blanke et al., 2009; Hsieh et al., 2010). *In vitro*, Thbs1 might reduce inflammation in human arthritic chondrocytes. Meanwhile, Thbs1 was chondroprotective for adipose stem cells. (Maumus et al., 2017). IL1RL1 (also known as ST2), a member of the IL-1 family, has a single ligand, IL33 (Griesenauer and Paczesny, 2017). The blockage of ST2 with a monoclonal drug reduces joint damage in mice with OA (He et al., 2020). Matrix metalloproteases (MMPs) are inhibited by TIMPs, and an imbalance in MMP13/TIMP1 causes abnormal degradation of the ECM, which is associated with the pathogenesis of OA (Arpino et al., 2015; Cabral-Pacheco et al., 2020). We considered that these ECM proteins respond to mechanical stimulation signals from LIPUS. But their role in the progression of OA, and the specific mechanisms of LIPUS regulation need further investigation. The selected ECM proteins were attractive candidates to be new molecular markers for determining the efficacy of LIPUS in OA. In addition, the intraarticular gene delivery of small molecular drugs such as THBS4 and THBS1 combined with LIPUS therapy could become a more efficient treatment for TMJOA.

With the exception of ECM proteins, changes in key factors of related pathways can predict the outcome of LIPUS. Previous studies have reported that LIPUS acts directly on the mechanoreceptor integrin on the cell membrane to promote FAK phosphorylation and then regulate chondrogenic differentiation (Takeuchi et al., 2008; Sang et al., 2020). In addition, integrins have been suggested to bind specifically to ECM substrates, which results in the formation of integrin adhesions and subsequently the initiation of downstream signaling (Hemler and Lobb, 1995). Focal adhesion occupied an essential position in integrin-mediated cell signaling transmission (Zhao and Guan, 2011). This study revealed that the KEGG pathway of focal adhesion was enriched, and multiple ECM proteins were identified as DEPs. LIPUS acted on specific proteins in the ECM such as THBS4 and THBS1, and activated the intracellular FAK-PI3K-AKT signaling pathway after binding to integrins. FAK is linked to the Regulation of actin cytoskeleton, Wnt signaling pathway and MAPK signaling pathway, according to the KEGG pathway map. After chemical signal transduction to the nucleus, the expression of related factors were regulated. Several functions such as cell motility, cell proliferation and cell survival were modulated.

The level of matrix production and degradation directly affects the homeostasis of the chondrocyte internal environment (Shen et al., 2020). Condylar articular cartilage consists of one type of cell—chondrocytes. Chondrocytes are wrapped in self-secreted ECM (Burrage et al., 2006; Charlier et al., 2019) and collagen II and Acan are vital components of these cells (Nagase and Kashiwagi, 2003). Sox9 regulates chondrogenic differentiation in the early stages and activates specific markers such as Acan and collagen II (Pratta et al., 2003). Additionally, at the osteochondral level, neovascularization worsens the inflammatory state within the joint, which accelerates destruction of the tissue structure (Mapp and Walsh, 2012). HIF-1α is unstable and rapidly degraded under normoxic conditions. However, it is stabilized in hypoxic environments and enters the nucleus, where it activates vascular endothelial growth factor (VEGF) expression (Salceda and Caro, 1997). Angiogenesis accelerates cartilage degradation and interacts with invading nerves, which causes pain in patients with OA (Roy et al., 2006; Baek et al., 2018). When arthritis occurs, angiogenesis, inflammation and hypoxia within the cartilage mutually reinforce each other. The results of this study demonstrated that LIPUS stimulation can increase ECM synthesis, alleviate the matrix degradation and reduce the degree of vascularization caused by hypoxia.

In conclusion, proteomic technologies can provide new insight into the pathogenesis and diagnosis of TMJOA. However, the progress in evaluating the therapeutic effects of LIPUS interventions remains relatively scarce. In this study, some ECM proteins (e.g., THBS4, Thbs1, IL1RL1, TIMP1, etc.) associated with the efficacy of LIPUS on TMJOA were initially screened and are expected to be reliable biomarkers. In the future, we will purposely analyze and validate the differentially expressed factors identified based on the results of this proteomic study. Further exploration of the mechanistic pathways through which LIPUS plays a therapeutic role in condylar cartilage injury will be performed. This research provided a basis for the early translation of basic research results into clinical applications, calling for joint efforts between clinicians and researchers of this burgeoning field in temporomandibular joint osteoarthritis. The abbreviations are listed in Table 4.

**TABLE 4:** List of abbreviations.

Full name	Abbreviation
Low-Intensity Pulsed Ultrasound	LIPUS
Tandem Mass Tag	TMT
Temporomandibular Joint Disorder	TMD
Temporomandibular Joint Osteoarthritis	TMJOA
Hypoxia-Inducible Factor	HIF
Differentially Expressed Proteins	DEPs
Gene Ontology	GO
Kyoto Encyclopedia of Genes and Genomes	KEGG
Protein-Protein Interaction	PPI
Extracellular Matrix	ECM
Focal Adhesion kinase	FAK
Aggrecan	ACAN
Matrix metalloproteinase	MMP
Tissue Inhibitor of metalloproteinase	TIMP
Vascular Endothelial Growth Factor	VEGF
Thrombospondin	THBS
Glutamine synthetase	GLUL
Liquid Chromatography-Tandem Mass Spectrometry	LC-MS/MS
False Discovery Rate	FDR
Peptide Spectrum Matching	PSM
Biological Process	BP
Cellular Component	CC
Molecular Function	MF
Phenylmethanesulfonyl fluoride	PMSF
Protease Inhibitor Cockail	PIC
Polyvinylidene fluoride	PVDF
Electrochemiluminescence	ECL
Tricarboxylic Acid Cycle	TCA

## Data Availability

The datasets presented in this study can be found in online repositories. The names of the repository/repositories and accession number(s) can be found below: http://www.proteomexchange.org/, PXD026677.
