# Supplementary material for: The Attenuating Effect of Low-Intensity Pulsed Ultrasound on Hypoxia-Induced Rat Chondrocyte Damage in TMJ Osteoarthritis Based on TMT Labeling Quantitative Proteomic Analysis
Source: Front Pharmacol. 2021 Dec 14;12:752734. doi: 10.3389/fphar.2021.752734 (PMC8712703; doi:10.3389/fphar.2021.752734)
Supplement: Supplementary file 1 [file DataSheet1.docx]

Supplementary Material

## Supplementary Figures


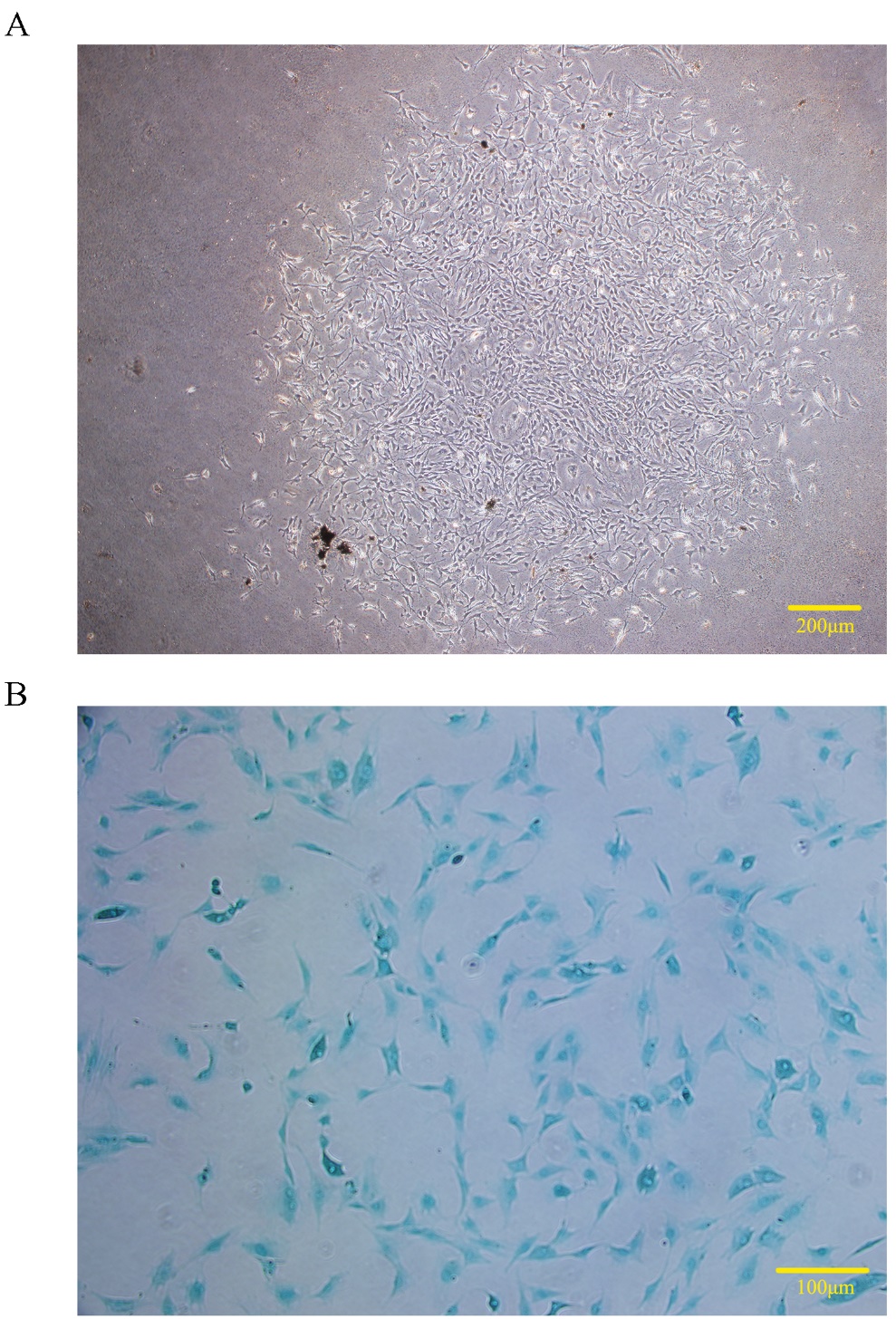


**Supplementary Figure 1.** Culture of primary chondrocytes (**A**) and Alcian blue staining identification of third-generation chondrocytes (**B**).


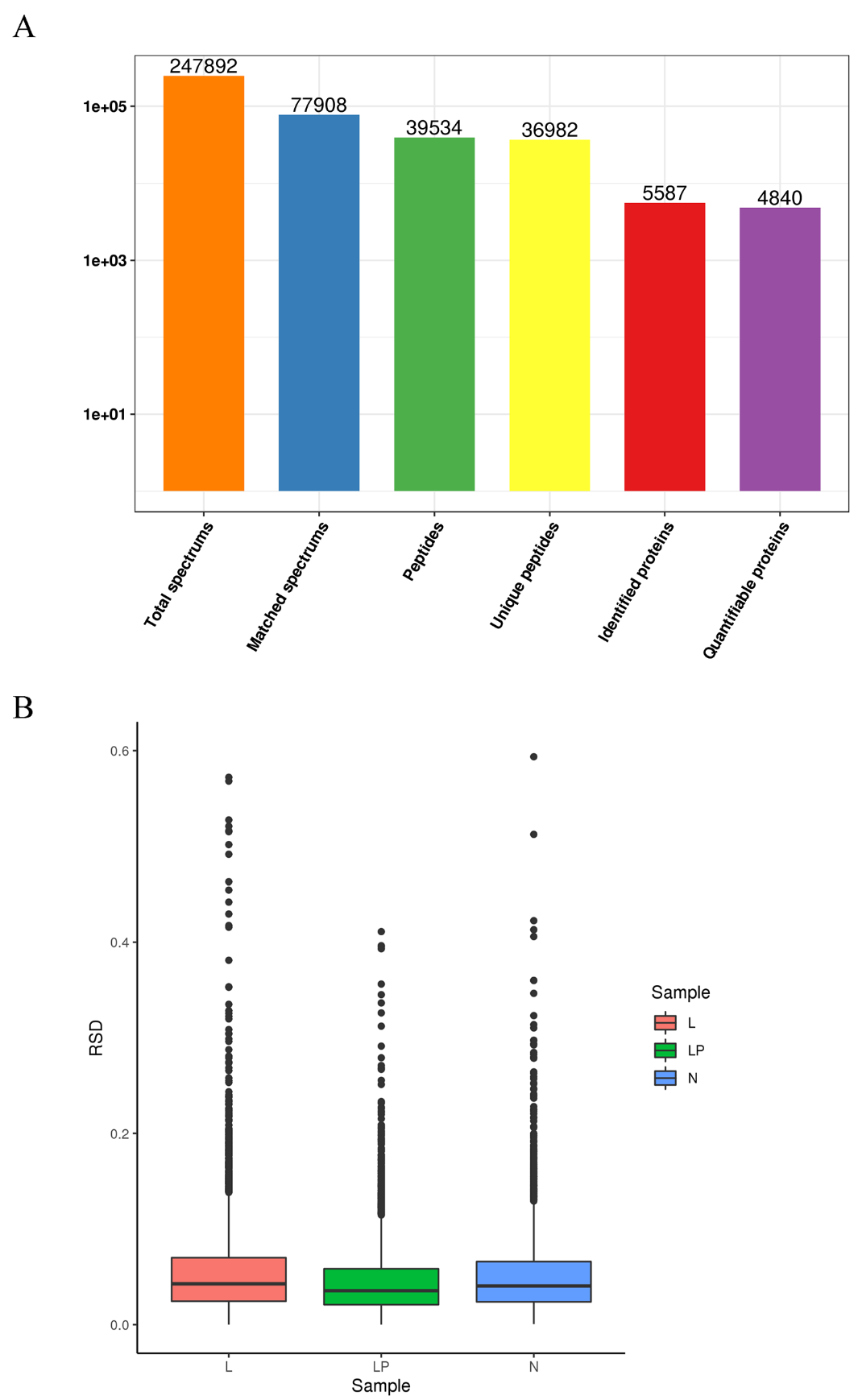


**Supplementary Figure 2.** Basic statistics of the mass spectrometry analyses (**A**). RSD boxplot showing the protein quantification between replicate samples of the N, L and LP groups (**B**).


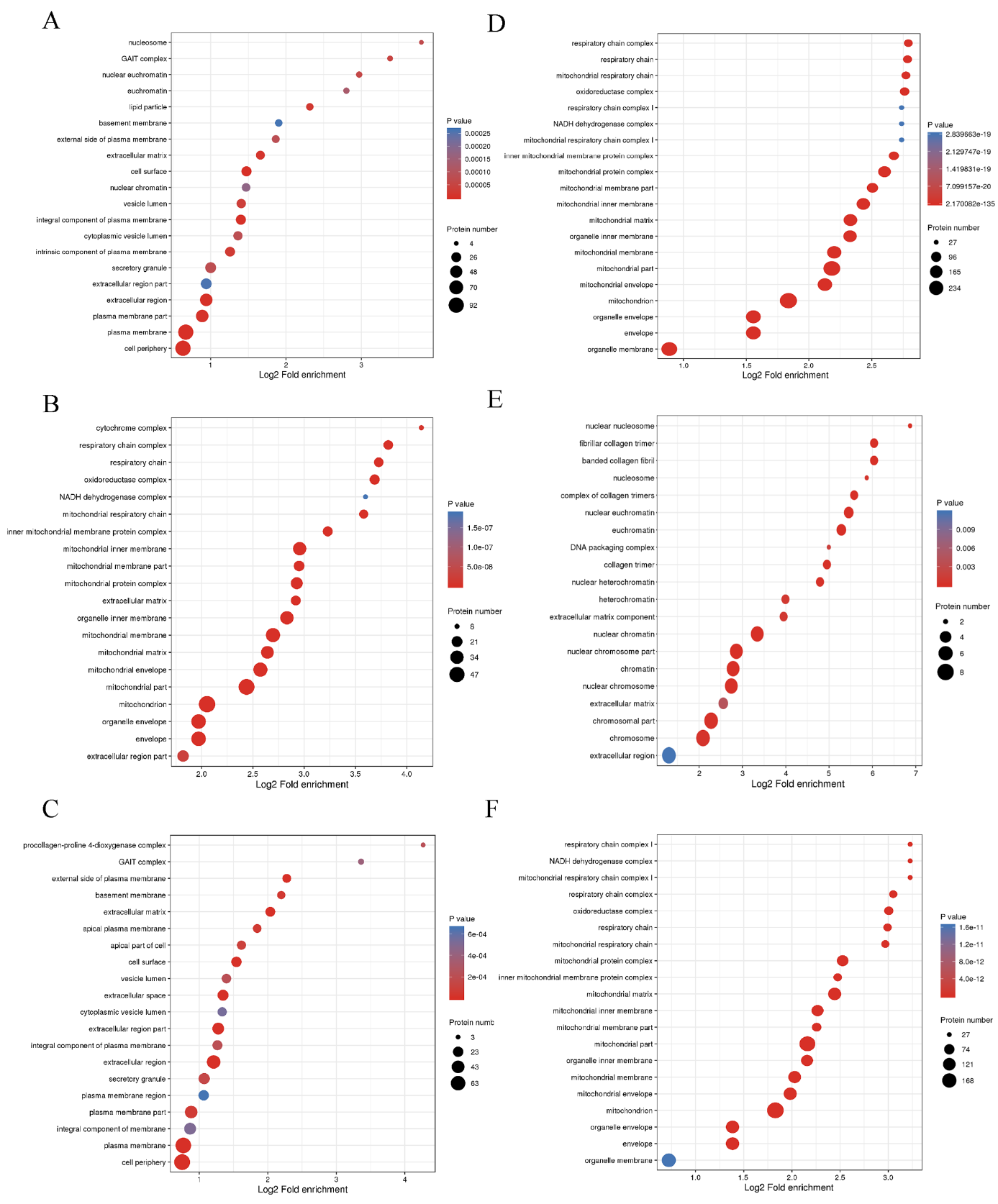


**Supplementary Figure 3.** GO enrichment analyses of DEPs. CC enrichment of upregulated DEPs between the L/N (**A**), LP/L (**B**) and LP/N (**C**) groups and downregulated DEPs between the L/N (**D**), LP/L (**E**) and LP/N (**F**) groups.


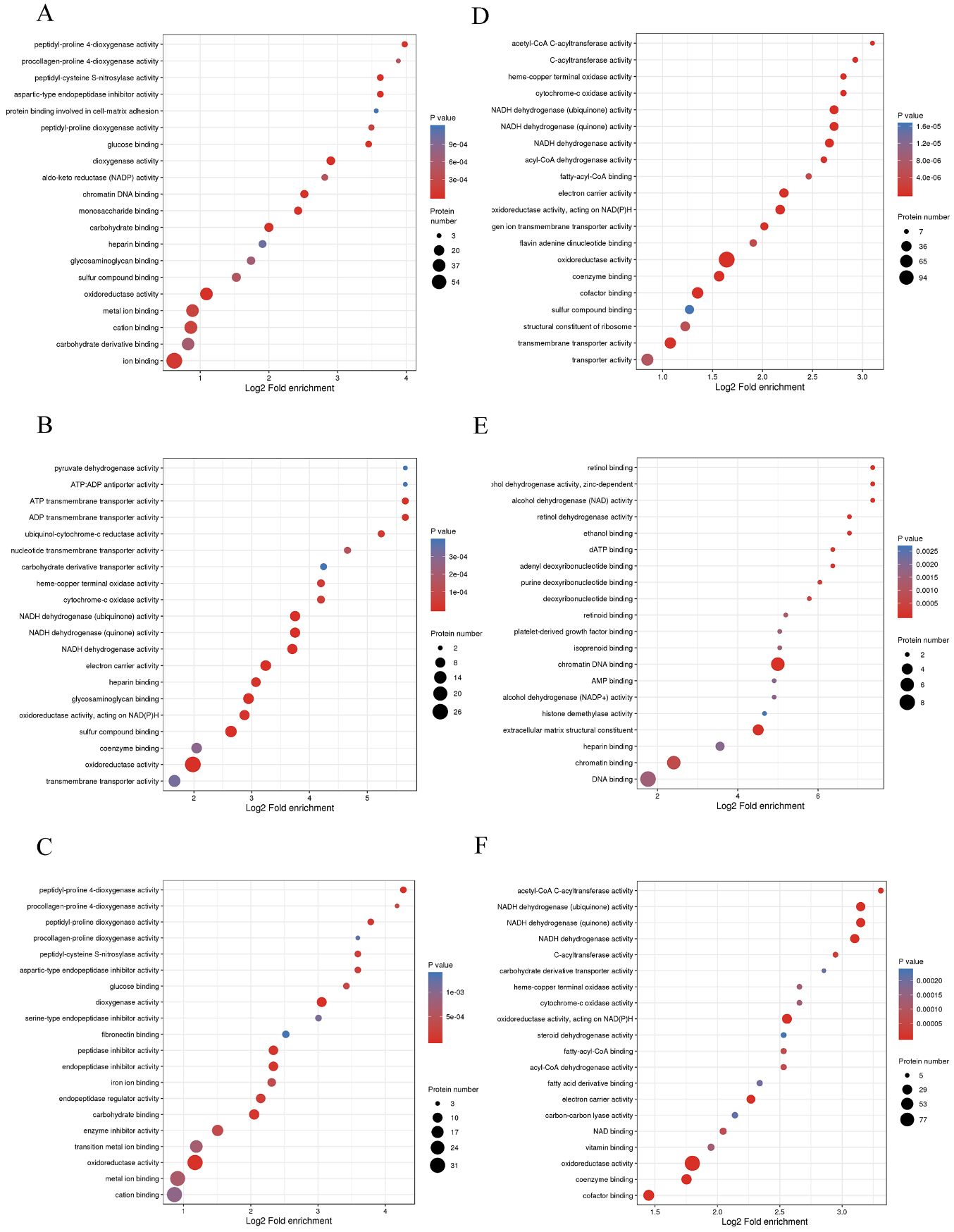


**Supplementary Figure 4.** GO enrichment analyses of DEPs. MF enrichment of upregulated DEPs between the L/N (**A**), LP/L (**B**) and LP/N (**C**) groups and downregulated DEPs between the L/N (**D**), LP/L (**E**) and LP/N (**F**) groups.


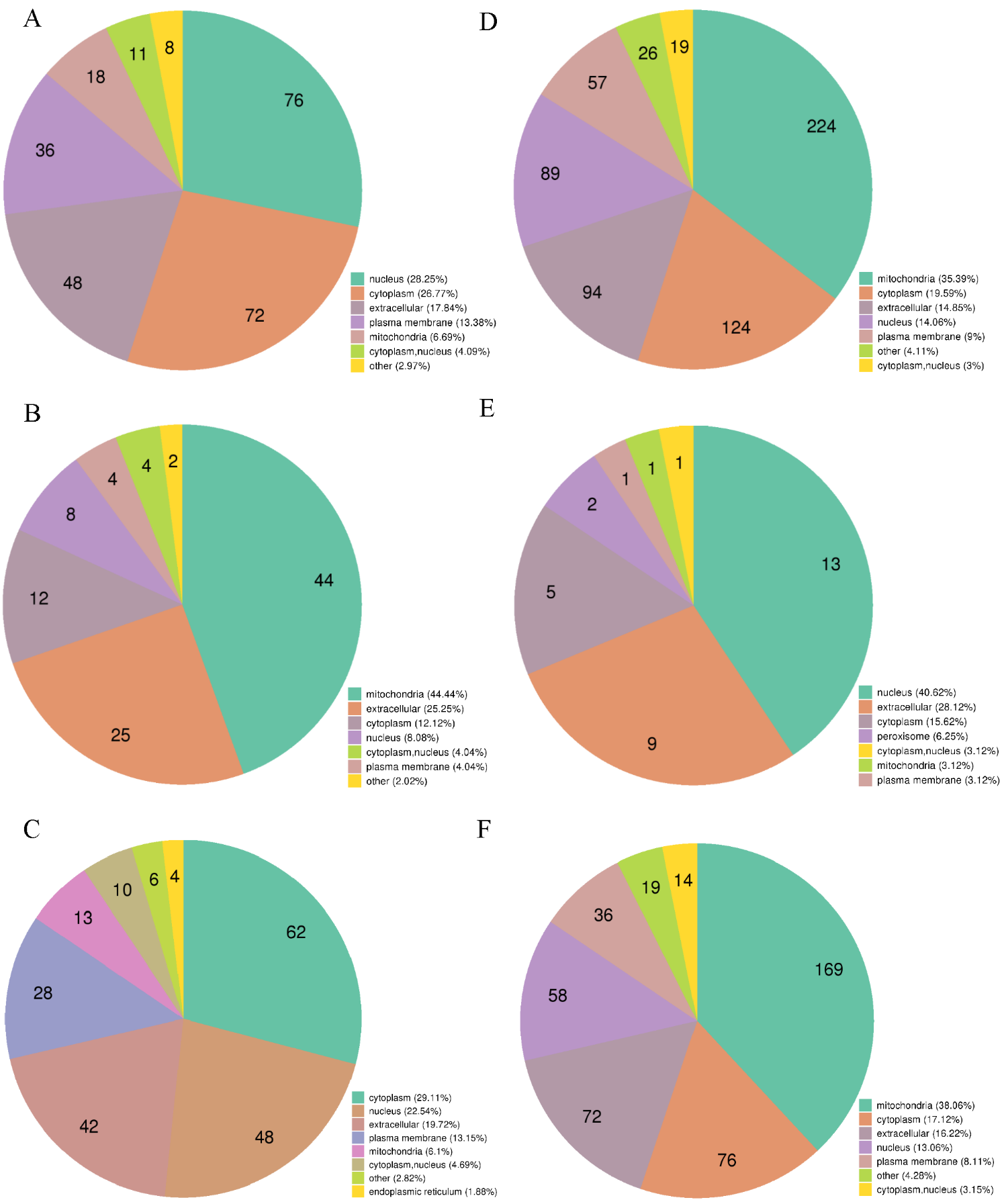


**Supplementary Figure 5.** Subcellular localization of upregulated DEPs between the L/N (**A**), LP/L (**B**) and LP/N (**C**) groups and downregulated DEPs between the L/N (**D**), LP/L (**E**) and LP/N (**F**) groups.
